# Integration of the landscape of fear concept in grassland management: An experimental study on subtropical monsoon grasslands in Bardia National Park, Nepal

**DOI:** 10.1002/ece3.70098

**Published:** 2024-08-01

**Authors:** Shyam Kumar Thapa, Joost F. de Jong, Anouschka R. Hof, Naresh Subedi, Yorick Liefting, Herbert H. T. Prins

**Affiliations:** ^1^ National Trust for Nature Conservation Lalitpur Nepal; ^2^ Zoological Society of London, Nepal Office Kathmandu Nepal; ^3^ Wildlife Ecology and Conservation Group Wageningen University and Research Wageningen The Netherlands; ^4^ Animal Sciences Group Wageningen University Wageningen The Netherlands

**Keywords:** habitat, herbivores, predation risk, predators, prey, tigers

## Abstract

The ‘landscape of fear’ concept offers valuable insights into wildlife behaviour, yet its practical integration into habitat management for conservation remains underexplored. In this study, conducted in the subtropical monsoon grasslands of Bardia National Park, Nepal, we aimed to bridge this gap through a multi‐year, landscape‐scale experimental investigation in Bardia National Park, Nepal. The park has the highest density of tigers (with an estimated density of ~7 individuals per 100 km^2^) in Nepal, allowing us to understand the effect of habitat management on predation risk and resource availability especially for three cervid species: chital (*Axis axis*), swamp deer (*Rucervus duvaucelii*) and hog deer (*Axis porcinus*). We used plots with varying mowing frequency (0–4 times per year), size (ranging from small: 49 m^2^ to large: 3600 m^2^) and artificial fertilisation type (none, phosphorus, nitrogen) to assess the trade‐offs between probable predation risk and resources for these cervid species, which constitute primary prey for tigers in Nepal. Our results showed distinct responses of these deer to perceived predation risk within grassland habitats. Notably, these deer exhibited heightened use of larger plots, indicative of a perceived sense of safety, as evidenced by the higher occurrence of pellet groups in the larger plots (mean = 0.1 pellet groups m^−2^ in 3600 m^2^ plots vs. 0.07 in 400 m^2^ and 0.05 in 49 m^2^ plots). Furthermore, the level of use by the deer was significantly higher in larger plots that received mowing and fertilisation treatments compared to smaller plots subjected to similar treatments. Of particular interest is the observation that chital and swamp deer exhibited greater utilisation of the centre (core) areas within the larger plots (mean = 0.21 pellet groups m^−2^ at the centre vs. 0.13 at the edge) despite the edge (periphery) also provided attractive resources to these deer. In contrast, hog deer did not display any discernible reaction to the experimental treatments, suggesting potential species‐specific variations in response to perceived predation risk arising from management interventions. Our findings emphasise the importance of a sense of security as a primary determinant of habitat selection for medium‐sized deer within managed grassland environments. These insights carry practical implications for park managers, providing a nuanced understanding of integrating the ‘landscape of fear’ into habitat management strategies. This study emphasises that the ‘landscape of fear’ concept can and should be integrated into habitat management to maintain delicate predator–prey dynamics within ecosystems.

## Graphical abstract

An aerial view of experimental plots in one of the open grasslands locally known as ‘Bagauraha Phanta’ in Bardia National Park, Nepal.

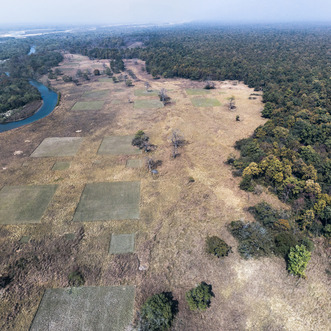

## 1 INTRODUCTION

The abundance and distribution of herbivores are often affected by predators in a system (Blumstein et al., 2006; Cherry et al., 2015; Kuijper et al., 2013; Wang et al., 2009). The ‘landscape of fear’ concept is a useful framework to understand how spatial variation in the mere risk of predation (not predation itself) influences prey behaviour and ecosystem processes (Fortin et al., 2005; Hernández & Laundré, 2005; Hof et al., 2012; Kohl et al., 2018; Laundré et al., 2010; le Roux et al., 2018; Lima & Dill, 1990; Valeix et al., 2010; Wheeler & Hik, 2014). However, there has been little experimentation with manipulating (perceived) predation risk to investigate whether the concept can purposefully be used for wildlife management (Ford et al., 2014; Gaynor et al., 2021; le Roux et al., 2018).

Within nature reserves, much effort in wildlife management is typically devoted to large mammalian herbivores (Forbes et al., 2019; Gaynor et al., 2019; Goheen et al., 2018). Given the ‘landscape of fear’, the behaviour of herbivores is steered by carnivores (Gaynor et al., 2019, 2021; le Roux et al., 2018). Management interventions may not reach intended objectives if the indirect effect of predation pressure is not accounted for. For example, herbivores may perhaps not or marginally use managed areas (e.g. mineral lick sites, mowed areas, fertilised areas, water holes, burned areas) if the perceived predation risk is high (Creel et al., 2005; Fortin et al., 2010; Gaynor et al., 2019; Laundré et al., 2010). However, despite the popularity of the landscape of fear concept, the effect of integration of, and accounting for (perceived) predation risk has as yet received little attention (but see Churski et al., 2021; Kuijper et al., 2013) and has not been investigated in the context of Asian subtropical grasslands inhabited by tigers (*Panthera tigris*).

The integration of the ‘landscape of fear’ concept into habitat management is crucial for predator–prey dynamics (Gaynor et al., 2021; Laundré et al., 2014). Habitat management can alter predator–prey dynamics by either favouring predators or prey. For instance, creating open space may reduce risk perception for prey (le Roux et al., 2018), but such interventions may also influence the hunting success rate of ambush predators like tigers (Karanth & Sunquist, 2000; Sunquist, 2010). Furthermore, herbivores may avoid managed habitats if they perceive them as too risky (Hebblewhite & Merrill, 2009; Hernández & Laundré, 2005), and hence management interventions may turn out to be fruitless and may have (unforeseen) cascading effects (Gaynor et al., 2019). The landscape of fear concept can inform habitat management interventions that optimise the trade‐off between risk and resources for herbivores (Hernández & Laundré, 2005; Laundré et al., 2010).

Herbivores are constrained by both top‐down (predation) and bottom‐up (food limitation) forces (Hopcraft et al., 2010; Le Roux et al., 2019) and their survival and fitness depend largely on their ability to optimise foraging benefits (Clinchy et al., 2013; Hebblewhite & Merrill, 2009; Wirsing et al., 2007). The number of tigers in Nepal has increased from an estimated 121 individuals in 2010 to 355 in 2022 (DNPWC & DFSC, 2022). Most tigers occur in national parks that are situated in the subtropical belt along the foothills of the Himalayas, which is the Terai in Nepal. With the increasing number of tigers within an otherwise unvarying area, the encounter frequency between predator and prey must increase, which makes it likely that individuals of the prey species become increasingly wary (Gaynor et al., 2019) and thus rely more and more on escape and avoidance tactics (Cromsigt et al., 2013; Lima & Dill, 1990). These antipredator responses often come at the cost of time spent on other essential activities such as foraging (Lima & Bednekoff, 1999; Say‐Sallaz et al., 2019). This can lead to a decrease in their performance (Clinchy et al., 2013) and ultimately affect their population dynamics (Chamaillé‐Jammes et al., 2019).

The primary prey species of the tigers in Nepal consist of muntjac (*Muntiacus vaginalis*), hog deer (*Axis porcinus*), chital (*Axis axis*), swamp deer (*Rucervus duvaucelii*) and sambar (*Rusa unicolor*) besides wild boar (*Sus scrofa*) (Lamichhane et al., 2019; Upadhyaya et al., 2018). Large body‐sized prey such as gaur (*Bos gaurus*) and nilgai (*Boselaphus tragocamelus*) only occur in relatively low densities (DNPWC & DFSC, 2022). Because these small‐ and medium‐sized deer forage mostly on grasslands (Moe & Wegge, 1994; Wegge et al., 2006) and require high‐quality forage to meet their nutritional requirements for survival (Ahrestani et al., 2012; Thapa et al., 2021), their foraging often translates into discernible vegetation patterning (Ford et al., 2014; Schmitz, 2008; Schmitz et al., 2004) as predation risk has the potential to alter or modify herbivores' foraging patterns (Hebblewhite & Merrill, 2009; Hernández & Laundré, 2005).

**FIGURE 1:**
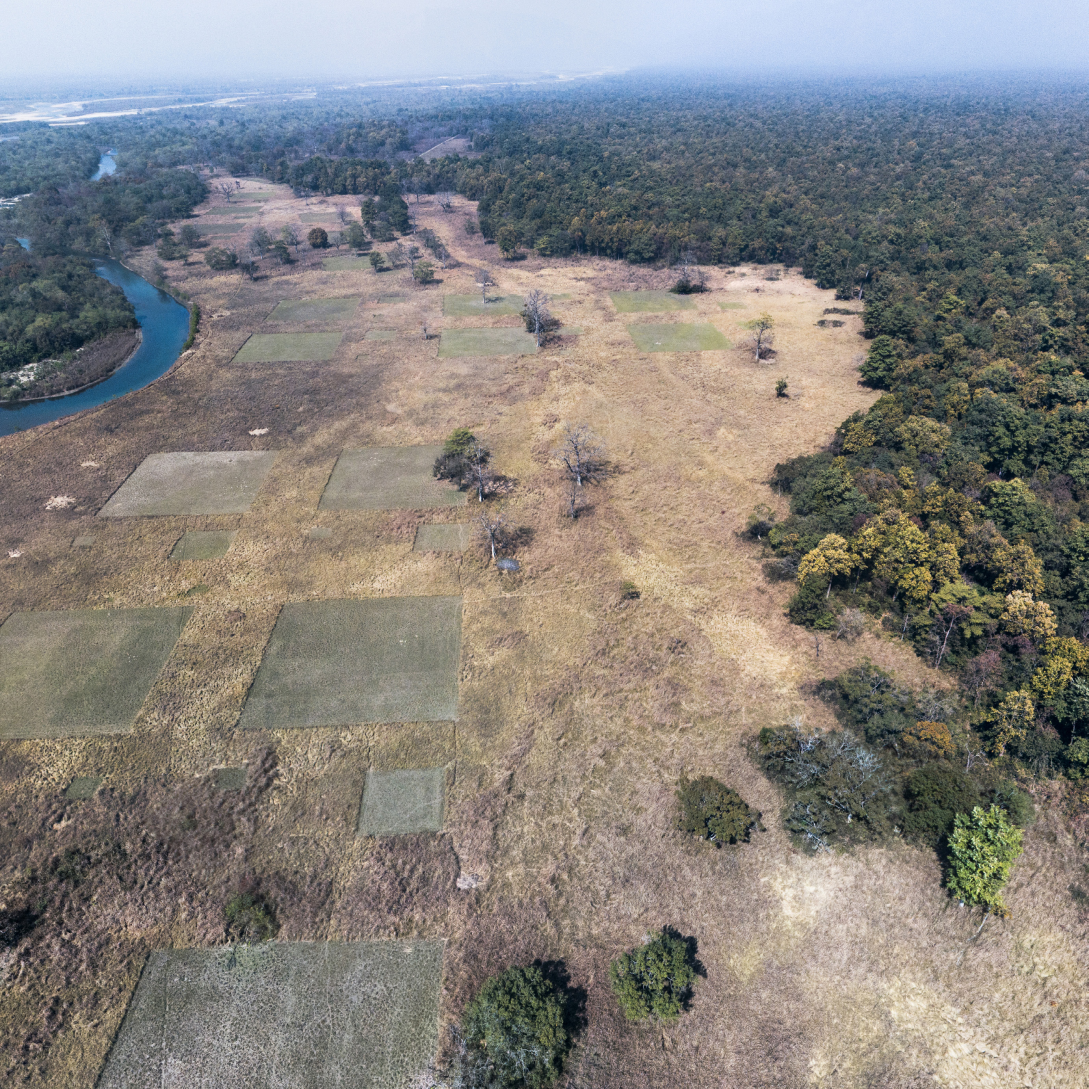
An aerial view of experimental plots in one of the open grasslands locally known as ‘Bagauraha Phanta’ in Bardia National Park. A landscape with clearly distinguishable plots of different sizes with short grasses and surrounding tall grasses creates gradients of predation risk. The grassland is frequently visited by tigers and small‐ and medium‐sized deer.

Here, we did a landscape scale experiment in a national park, in an area with a high tiger density, to explore whether deliberate integration and accounting for perceived predation risk affects the effectiveness of wildlife management interventions. By simultaneously examining the effect of altering resource quality (primarily through mowing and artificial fertilisation) and manipulating predation risk (primarily by creating open areas of different sizes—plot size) on the level of use of the managed grassland by three cervids (small hog deer—with an average weight of ~40 kg, medium‐sized chital of ~50 kg and swamp deer of ~150 kg), we explore the applicability of the ‘landscape of fear’ concept in habitat management. We predicted that the size of the experimental plots and grass heights within the plots would be important factors influencing habitat selection. Both the size of plots and grass height create gradients in perceived predation risk by altering visibility, detection probability and fleeing ability (Laundré et al., 2010; le Roux et al., 2018). To our knowledge, this is the first landscape‐level empirical study from South Asia where we incorporate the concept of ‘landscape of fear’ into habitat management (Figure 1). Here, we report on a series of experiments that were executed to incorporate the ‘landscape of fear’ concept into grassland management in the subtropical monsoon grasslands in Nepal. Our study provides novel insights into the applicability of the ‘landscape of fear’ concept in grassland management and contributes to the conservation of predator and prey species in the ecosystem.

## 2 MATERIALS AND METHODS

### 2.1 Study site

**FIGURE 2:**
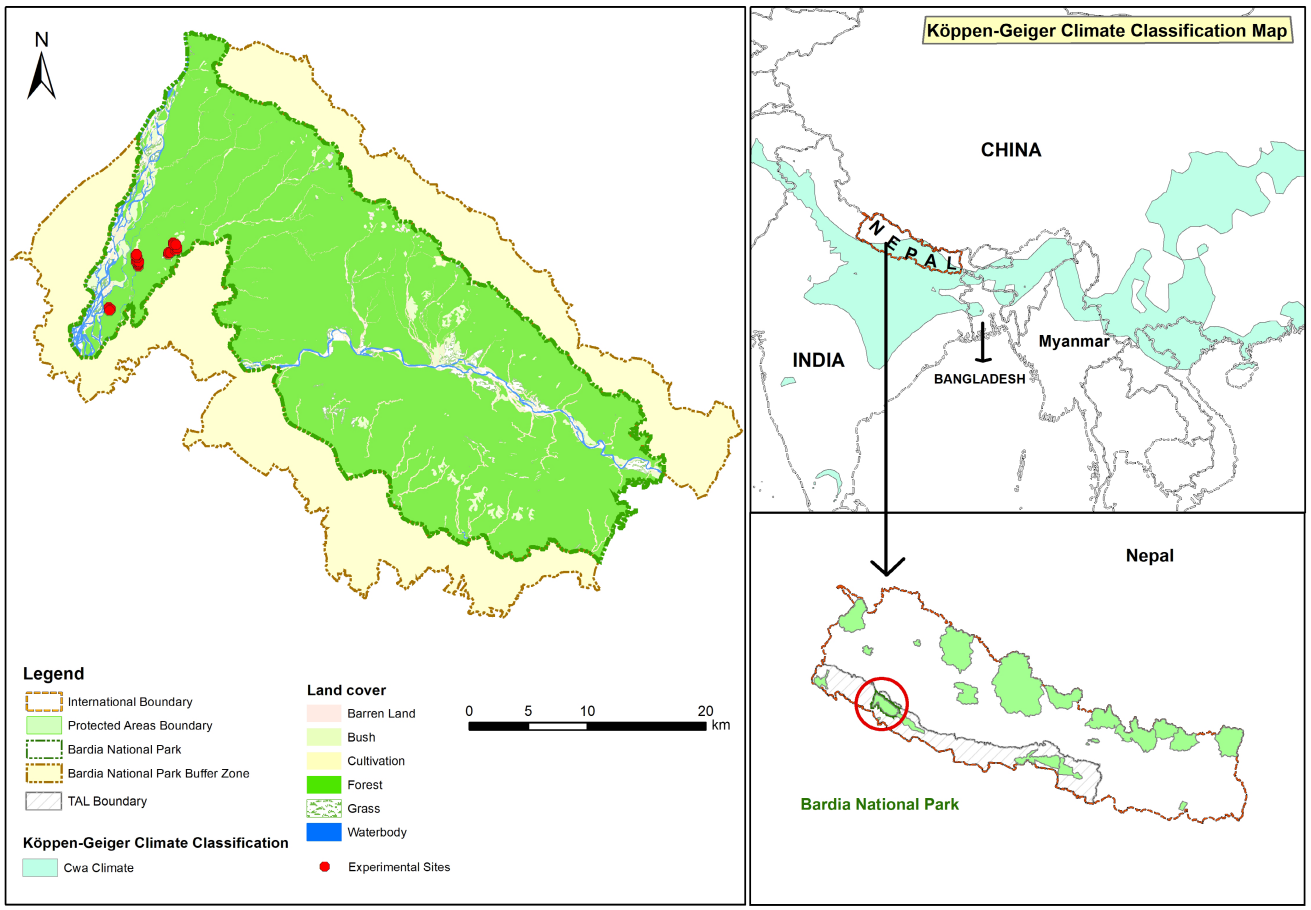
Experimental plots were established within the subtropical monsoon grasslands located in the core area of Bardia National Park (NP). The Barida NP is situated within the Terai Arc Landscape of Nepal (bottom‐right inset map) and holds the highest density of tigers in Nepal. The park falls within the Cwa climate region, characterised by a monsoon‐influenced humid subtropical climate according to the Köppen‐Geigen classification (light blue area on the top‐right inset map).

We conducted our study in the subtropical monsoon grasslands located in the core area of Bardia National Park (Bardia NP) of Nepal (Figure 2). The area falls within the Cwa‐climate monsoon‐influenced humid subtropical climate region, based on Köppen‐Geigen climate classification (Chen & Chen, 2013). Bardia NP is one of the largest national parks within the Terai Arc Landscape of Nepal covering an area of 968 km^2^ (centre of the park at 28°23′ N, 81°30′ E). Bardia NP has monsoon (June–September), winter (October–February) and summer (March–May) seasons. The mean monthly temperature ranges from a minimum of 10°C to a maximum of 45°C. The park receives a mean annual rainfall of ~1700 mm (Thapa et al., 2022).

Bardia NP consists of subtropical vegetation with riverine forest, riverine floodplain grasslands along the two major rivers (Karnali and Babai rivers), *sal* (*Shorea robusta*) forest with interspersed grasslands, and mixed hardwood forests (Dinerstein, 1979).

The park holds the highest density of tigers in Nepal with an estimated density of ~7 individuals 100 km^−2^ and an estimated prey density of ~90 individuals km^−2^ (DNPWC & DFSC, 2022). Chital, at present, is the most abundant primary prey in the system (Upadhyaya et al., 2018) with a reported density of ~45 deer km^−2^ (DNPWC & DFSC, 2022) after larger prey species [arna (*Bubalus arnee*) and gaur] went extinct (Jhala et al., 2021) or got reduced to fewer than a handful, for example, nilgai (Wegge et al., 2009). Tigers exclusively reside within the protected area of the park (Figure 2) and the studied grassland sites are within the area with the highest density of tigers in the park (Figure S1, DNPWC & DFSC, 2022). Thus, deer in Bardia NP live under high predation risk and most of the direct and indirect predator–prey interactions occur within the protected area of the park as a dispersal of the animals is limited by surrounding farmland and settlements within the buffer zone (Figure 2). The assemblage of predators and prey species in Bardia NP offers an ideal situation to experimentally test if herbivores can be successfully managed by manipulating predation risk.

The tiger is of main concern because of its threatened status on the IUCN Red List and the Government of Nepal's goal to maintain its population at the recently achieved high numbers without aggravating the precarious status quo with villagers living in surrounding settlements by reducing incidents of human–tiger conflicts.

### 2.2 Experimental design

The experimental sites were situated in the western section of the park in the three disjointed patches of open grassland that are interspersed within *sal* forests (Figure 2). We set up multi‐year large‐scale experimental plots (*n* = 189, Table 1) in the protected area of Bardia NP, thus, giving us unique opportunities to test empirically the applicability of the ‘landscape of fear’ concept in grassland management for ungulates. We outlined 189 plots in three disjoined patches of open grassland where we manipulated resources and the risk of predation. The distance between the patches was between 1 and 2 km. These patches were at the same topographic positions in the landscape, comprised of similar vegetation (Thapa et al., 2021), and are frequently used by medium‐sized swamp deer and chital and smaller hog deer (Thapa et al., 2022). We solely considered these three deer species for this study as the other species are only present in small numbers.

**TABLE 1:** Total number of experimental plots with different levels of treatments.

Plot size	No mowing			2 times mowing			4 times mowing			Total
	No fertilisation	Nitrogen	Phosphorus	No fertilisation	Nitrogen	Phosphorus	No fertilisation	Nitrogen	Phosphorus	
49 m^2^	7	7	7	7	7	7	7	7	7	63
400 m^2^	7	7	7	7	7	7	7	7	7	63
3600 m^2^	7	7	7	7	7	7	7	7	7	63
Total	21	21	21	21	21	21	21	21	21	189

*Note*: The plots were established in three grassland patches within the core area of the Bardia National Park.

We mowed grasses and spread chemical fertilisers in the experimental plots to attract herbivores to the plots. Because we are interested in trade‐offs between risks and resources, we used chemical fertilisers together with mowing to increase the quality of forage (Schroder, 2021; Thapa et al., 2023), which eventually created an attractive environment for grazing.

### 2.3 Experimental plot set‐up

The experimental design incorporated three treatment factors: mowing; artificial fertilisation, and plot size and each treatment factor had three levels. There were a total of seven replications spread over three sites, forming a complete design with 189 experimental plots (Table 1). The scale of the landscape did not allow for more plots. We laid out square plots of different sizes (3600; 400 and 49 m^2^) in each replication.

**FIGURE 3:**
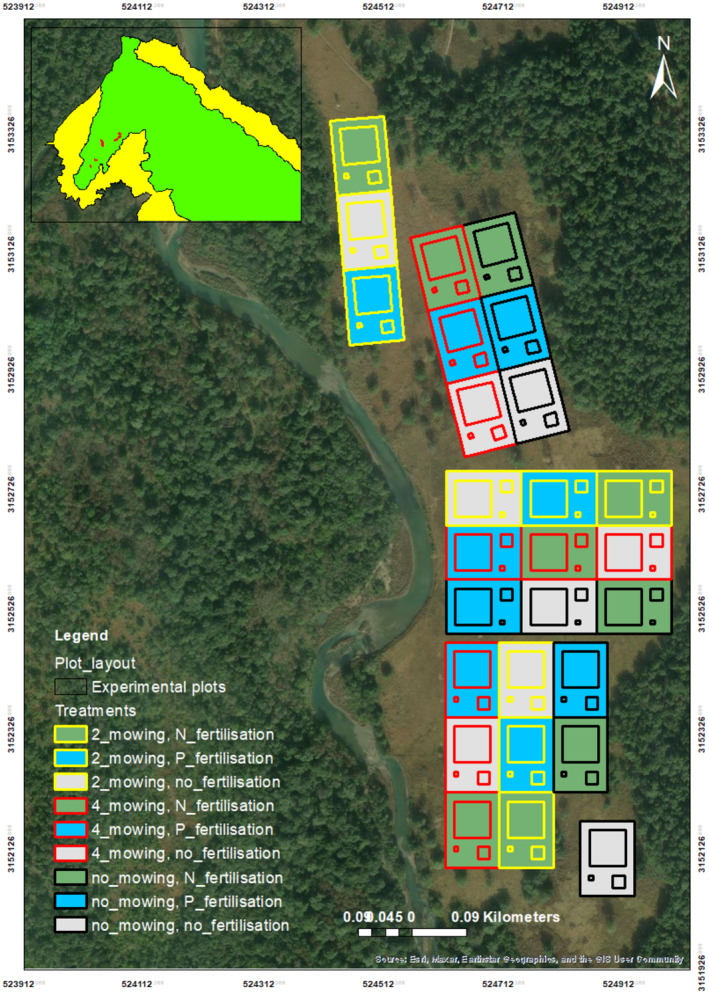
Complete set (three replications) of experimental design in one of the locations (Baghaura phanta, *n* = 81) within Bardia NP. One replication unit consisted of 27 plots. There are a total of 7 replications within three sites. The colour of the borderline of the square plots indicated the types of mowing treatment received by respective plots—black borderline for no mowing, yellow for two times mowing and red for four times mowing per year. Likewise, filled colours within square plots represent types of fertilisation treatment received by respective plots—green for nitrogen fertilisation, blue for phosphorous, and grey for no fertilisation. The size of the square represents either 3600 or 400 or 49 m^2^ plots. Experimental plots were established within the protected area of Bardia NP (red dots in the light green area in the top‐left inset map).

Plots received different levels of mowing (no mowing, two times mowing and four times mowing per year) and fertilisation treatments (nitrogen fertilisation, phosphorus fertilisation or no fertilisation) at random. Each complete replicate comprised nine plots of 3600 m^2^, nine plots of 400 m^2^ and nine plots of 49 m^2^, totalling 27 plots (Figure 3). Within the nine plots of each size, three received four times mowing, three received two times mowing and three received no mowing. Similarly, within each size category, three plots received no fertilisation, three received nitrogen fertilisation and three received phosphorus fertilisation. This arrangement ensured a total of 27 plots per replicate, with variations in mowing and fertilisation treatments across the different plot sizes (see Figure 3). We determined the level of mowing and fertilisation treatments for each plot using computer‐generated random numbers. Additionally, to maintain isolation, a buffer area of at least 15 m was maintained between plots and between replicates (Figure 1).

The mowing treatments were applied in 2019 and 2020. Tall dense grasses (~1.5 to ~3 m tall) were mowed at a height of around 5 cm from the ground. Grasses were mown in January/February and August/September in plots that received two times mowing treatments. Grass vegetation was mown in January/February, April/May, August/September and November/December in plots that received the four times mowing treatment. We did not cut grasses from the plots that received no mowing treatment. We removed aboveground biomass after mowing from the mown plots. We applied either urea (46% N) or single superphosphate (SSP with P_2_O_5_ 16%) in those plots that were labelled to receive respective fertilisers (Table 1). We spread fertilisers three times (2018, 2019 and 2020) to stimulate nutrient‐rich regrowth (Schroder, 2021). The first two applications equated to 15 g m^−2^ of urea and 15 g m^−2^ of SSP (in March 2018 and April 2019). We increased the load in the third application and spread 40 g m^−2^ of urea and 60 g m^−2^ of SSP in January 2020 because the low application of fertilisers in previous years resulted in a weak contrast between treated and non‐treated plots. We used plots that received no mowing and no fertilisation (*n* = 21, Table 1) as controls.

### 2.4 Data collection

#### 2.4.1 Pellet density to estimate the level of use by deer

To assess the level of use of the experimental plots by small‐ and medium‐sized deer (viz. hog deer, chital and swamp deer), we estimated pellet group density expressed as pellet groups.m^−2^. Pellet group count may not be the best method to quantify habitat selection for foraging, but it provides a reliable estimate for the level of use of the habitat (Cromsigt et al., 2009; Härkönen & Heikkilä, 1999; Månsson et al., 2011; Thapa et al., 2022). We distinguished pellet groups at the species level based on the pellet morphology (see Thapa et al., 2022) and recorded them separately. We used a 2 × 2 m frame to record pellet groups from sampling points in each experiment plot. Only pellet groups with five or more pellets were recorded and pellet groups with more than 75% of the pellets outside of the frame were not recorded. We surveyed approximately 2% of each plot area, except for 49 m^2^ plots, where we recorded pellet groups from one sampling point at the centre. In 400 m^2^ plots, we recorded pellet groups from two sampling points (one at the edge and one at the centre) and in 3600 m^2^ plots, we systematically laid out 21 evenly spaced sampling points (see Figure S2 for the spatial layout of sampling points). We recorded pellet groups in each plot (*n* = 189) monthly and used an average value per plot for seasonal comparisons. We also measured grass height for each plot within a 2 m × 2 m sampling frame and averaged it at the plot level.

The spatial layout of sampling points in 3600 m^2^ plots allowed us to measure and compare the pellet density at the edge and central (core) areas of the plots. For this, we considered 3600 m^2^ plots that received mowing and fertilisation treatments. Because of the predation risk, we considered it likely that the edge and central (core) area of the 3600 m^2^ plots were differentially used by the deer, with the possibility of aggregation of deer at the core area where they may feel safe and the likelihood of early detection of predators is also high. A resulting concentrated grazing at the centre may kick‐start a grazing lawn formation process (Thapa et al., 2023).

### 2.5 Data analyses

We used a linear mixed effect model (LMM) to compare pellet density (expressed as pellet groups.m^−2^) with different levels of treatments on plots. We used pellet group density to express the level of use of the plots by deer. First of all, we investigated the effect of plot size on the level of use by deer. For this, we used log_e_ transformed plot size as a fixed component and replication within the location as random intercepts in the model.

We assessed the effect of treatments (mowing, fertilisation and plot size) on the level of use through LMM. We analysed pellet density (expressed as pellet groups.m^−2^) as the dependent variable and treatments (mowing, fertilisation and plot size), seasons, species and their interactions as fixed factors in the model. Since the level of use of the habitat by deer differs with seasons (Moe & Wegge, 1994) and species of deer (Pokharel & Storch, 2016), we included these two terms in the model. We included vegetation height as a covariate in the model because vegetation height is an important factor that affects the visibility and detection probability of predators and hence the level of risk perception. As random effects, we had intercepts for replications within locations in the model.

We also examined the differences in pellet density (expressed as pellet groups.m^−2^) between the edge and centre (core area) of the experimental plots on a subset of the 3600 m^2^ plots (*n* = 42, Table 1) that received mowing (two and four times mowing) and fertilisation (no, nitrogen or phosphorus fertilisation). Pellet density was modelled with treatments (mowing, fertilisation and plot size), season, species, point (edge or centre of the plot) and their interactions as fixed components in LMM. We included replications within locations as a random factor in the model. Visual inspection of residual plots (histogram, normal probability plot, residuals vs. fitted values) from all the mixed models did not reveal any violation of the LMM assumptions viz., residuals were normally distributed, error terms were normally distributed, and no obvious deviations from normality were detected.

All statistical analyses were performed in R, version 4.1.0 (R Core Team, 2021). We used the ‘lme4’ package (Bates et al., 2015) for the LMMs. Post hoc multiple comparison tests were performed using the ‘emmeans’ package (Lenth et al., 2021) after the LMMs. All graphs were prepared using the ‘ggplot2’ package (Wickham, 2021).

## 3 RESULTS

### 3.1 Pellet density with respect to the spatial scale of the interventions

**FIGURE 4:**
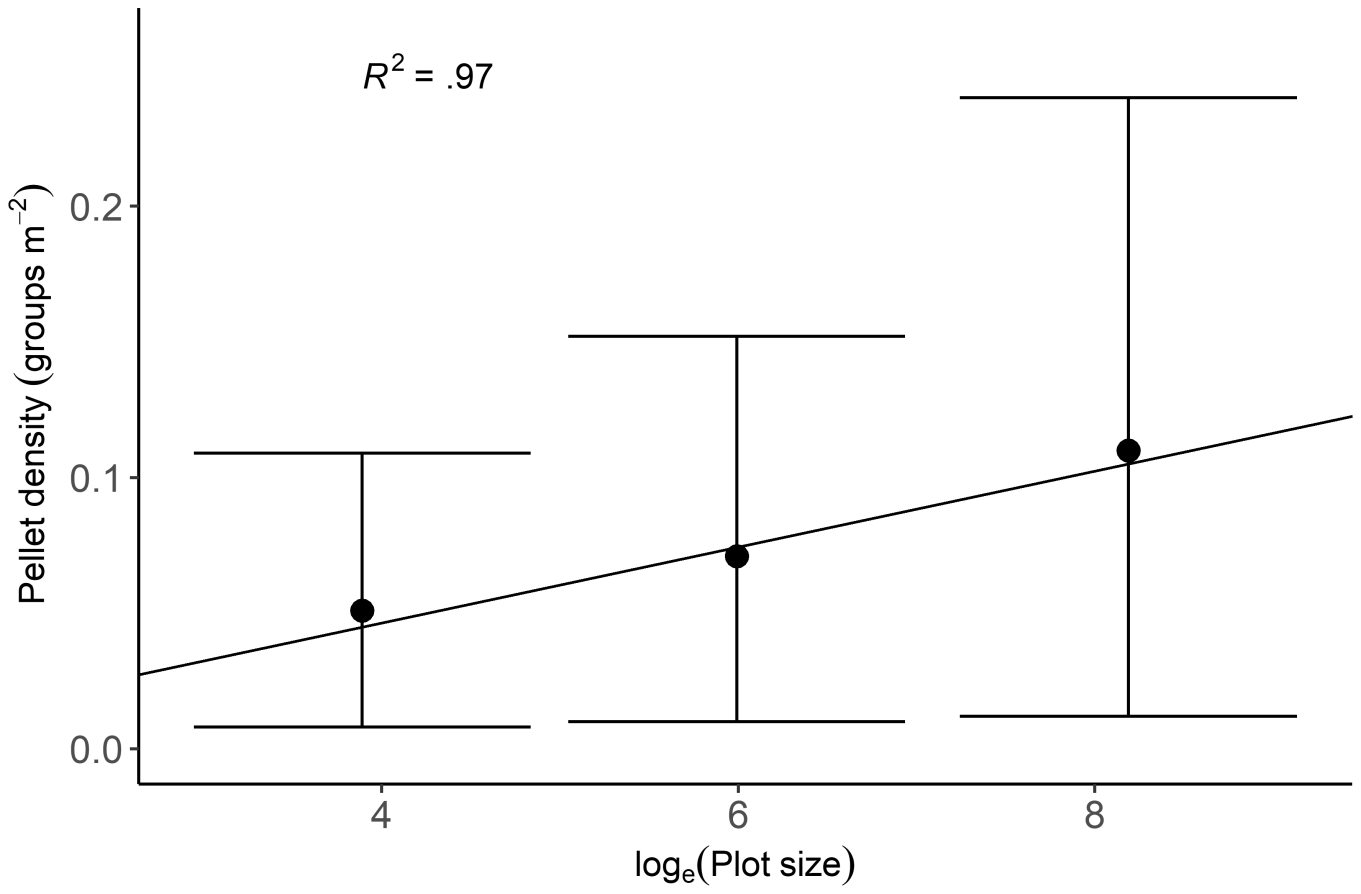
Pellet density (groups m^−2^) in different‐sized experiment plots expressed as log_e_ (plot size). Error bars represent 95% CI. The equation for the line is *y* = −0.0096 + 0.015 *x* and is obtained from an LMM with log_e_ (plot size) as a fixed factor. Here, *y* stands for pellet density, and *x* is the log_e_ transformed plot size.

We recorded twice the density of pellet groups in 3600 m^2^ plots [mean = 0.1 pellet groups m^−2^ (95% CI: 0.10–0.13)] as in 49 m^2^ plots [mean = 0.05 pellet groups m^−2^ (95% CI: 0.04–0.06)] and 1.5 times higher than in 400 m^2^ plots [mean = 0.07 pellet groups m^−2^ (95% CI: 0.06–0.08)]. The pellet density increased significantly (*F* = 64.99, *p* < .001) with log_e_ transformed plot sizes (Figure 4). Pellet density increased with a unit of 0.015 for every one unit increase in log_e_ transformed plot size.

### 3.2 Management interventions and pellet density

Pellet density varied in plots with respect to treatments: mowing, fertilisation, plot size and the interaction effects with species and season (Appendix A). Vegetation height had a significant effect on the level of use of the managed areas (*F* = 18.06, *p* < .001, Appendix A). Vegetation height decreased significantly with mowing (*F* = 154.95, *p* < .001, Appendix B) and had a significant effect on the level of use by the deer (*F* = 610.09, *p* < .001, Appendix C).

**FIGURE 5:**
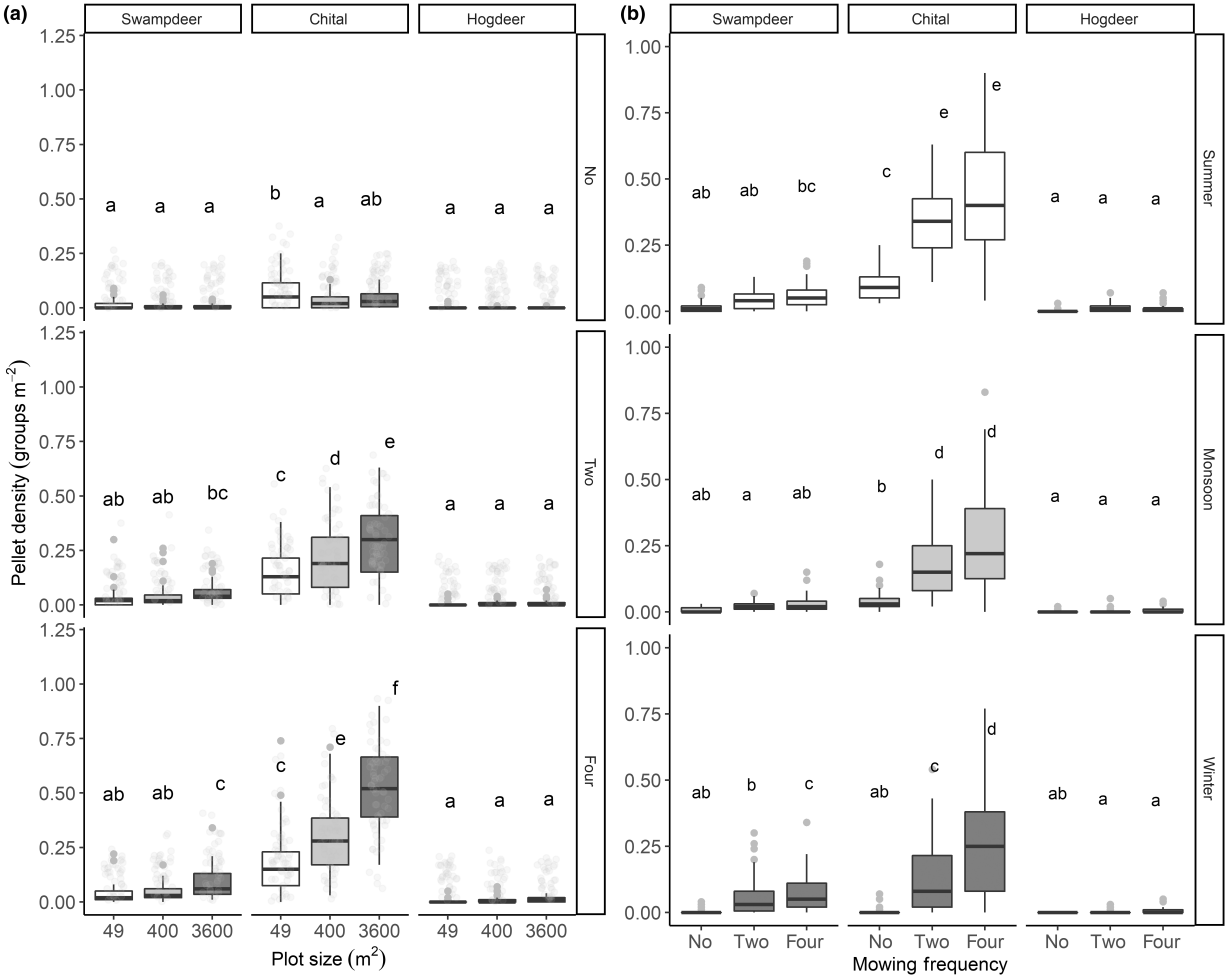
Level of use of the managed grasslands with respect to (a) Plot size, mowing and species; (b) mowing, season and species. The level of use was measured through pellet density (groups m^−2^) recorded in each experimental plot. Letters above each boxplot indicate a significant difference at alpha = .05, tested by estimated marginal means grouped by mowing after the LMM. Groups that share the same letter are not significantly different from each other.

At the species level, pellet groups of chital and swamp deer were higher in 3600 m^2^ plots than in 400 m^2^ or 49 m^2^ plots. In contrast, the pellet density of small hog deer did not differ significantly with plot size (Figure 5). Pellet density of chital and swamp deer was higher in the 3600 m^2^ plots that were mown four times (*F* = 50.12, *p* < .001; Figure 5a). The interaction effect of season and mowing was significant for chital, while the effect for swamp deer was significant only in winter (*F* = 9.83, *p* < .001; Figure 5b). On the contrary, the pellet density of hog deer did not differ significantly with mowing, plot size and season (Figure 5). Similarly, fertilisation had a significant effect on the level of use (*F* = 6.29, *p* = .002; Appendix A). Additionally, there was a significant interaction effect of plot, fertilisation and species on the level of use (*F* = 2.34, *p* = .02; Appendix A).

### 3.3 Pellet density at a fine scale

**FIGURE 6:**
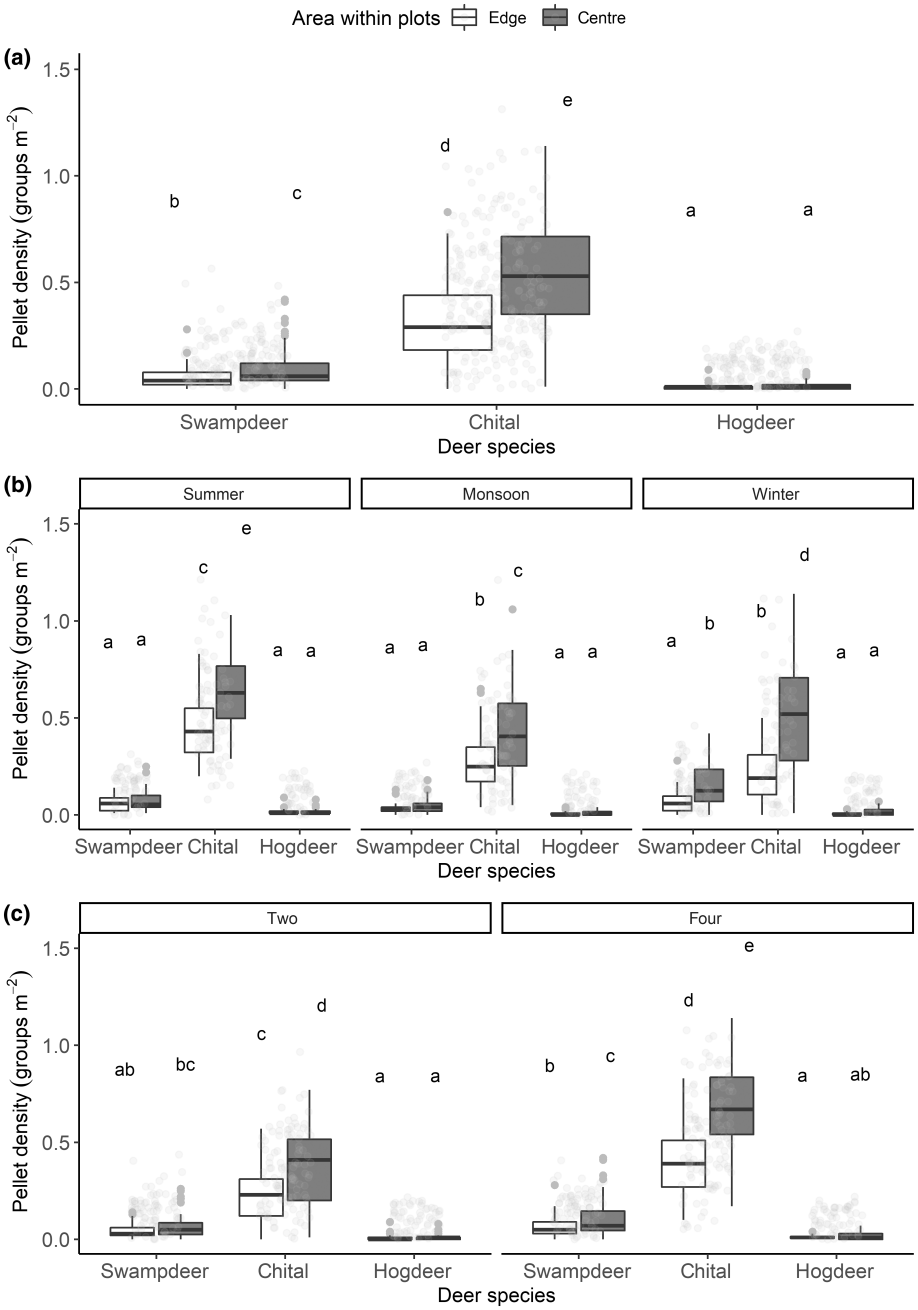
Level of use of the edge and centre areas of the plots in Bardia National Park differs with respect to (a) species, (b) season and (c) mowing. The white boxplot denotes the edge and the grey‐shaded boxplot represents a central (core) area of the plots. Letters above each boxplot indicate a significant difference at alpha = .05, tested by estimated marginal means grouped by point (area within plots: Centre and edge) after the LMM. Groups that share the same letter are not significantly different from each other.

At a finer scale, pellet density [mean = 0.21 (95% CI: 0.18–0.24)] was higher in the central (core) area of the 3600 m^2^ plot than in the edge area [mean = 0.13 (95% CI: 0.11–0.14)] of the plot (*F* = 171.55, *p* < .001). The interaction effect of mowing, species and area within the plot (point in the model, *F* = 9.73, *p* < .001, Appendix D), and the interaction effect of species, season and area within the plot (*F* = 3.13, *p* < .015, Appendix D) showed a significant effect on pellet density in plots. The pellet density of chital and swamp deer was significantly higher in the central (core) area of the plots than in the edge of the plots (Figure 6a). Chital's pellet groups in the core area of both two‐ and four‐times mown plots were significantly more than in the edge area of these plots (Figure 6b,c). Pellet groups of swamp deer were higher in the central area of four times mown plots only during winter (Figure 6b,c). In contrast, the pellet groups of small hog deer did not differ significantly between the edge and the centre irrespective of mowing and seasons (Figure 6).

## 4 DISCUSSION

Through a landscape‐scale experiment in the core area of Bardia NP, Nepal, we manipulated the quality of the forage (by mowing and fertilisation), modified the presumed risk perception of deer (by creating open areas of different sizes which we called plots) and quantified the response of small‐ and medium‐sized deer in terms of level of use through pellet group density. Our results showed that chital and swamp deer indeed selected managed larger grassland patches with short grasses over unmanaged patches and surrounding edges with tall grasses. We hypothesise that these deer feel safer in large plots with short grasses because of a higher likelihood of early detection of approaching tigers, and a better chance of escaping from predation than in small plots. Our study on this predator–prey system shows how habitat management affects the perceived predation risk and the resulting trade‐off between resource availability and safety, with insights relevant beyond our study area.

By removing tall grasses, deer use increased compared to unmanaged (un‐mown) plots, as previously demonstrated (le Roux et al., 2018; Moe & Wegge, 1997). Our study further establishes that the level of use is directly related to the extent of openness and grass height (visibility), while forage quality plays a lesser role. This finding aligns with previous studies that identified visibility (Wheeler & Hik, 2014), detection probability (Valeix et al., 2009), distance to refuge (Cresswell et al., 2010; Iribarren & Kotler, 2012) as critical determinants of predation risk perception and response in prey species (Gaynor et al., 2019).

Mowing treatment may lower predation risk by decreasing grass height and increasing visibility, while also enhancing forage quality, thereby creating attractive foraging ground for herbivores (Schroder, 2021; Thapa et al., 2023). Our results showed that herbivores tend to avoid risky areas even when those areas offer high‐quality forage. We found that large (3600 m^2^) plots, and within those large plots, core areas of the plots had a relatively high density of pellets of swamp deer and chital and a relatively low pellet density of hog deer. The metaphorical ‘landscape of fear’ is species‐specific (Hopcraft et al., 2010; Le Roux et al., 2019) and so is an integration of ‘landscape of fear’ in habitat management. However, a better term would be ‘landscape of risk’ as long as the parameters of the internal emotional states of the animals are unknown. A study on hunting success of female lions (*Panthera leo*) found that when the distance between a female lion and its prey was 20 m, most of the prey animals were able to escape predation (Elliott et al., 1977). The core areas of larger plots with short grasses likely offer deer a better chance of detecting approaching predators early and a greater likelihood of escape with the necessary flight distance (Cresswell et al., 2010; Stankowich & Coss, 2006). This could be a reason for the higher aggregation of deer in the core area of larger plots, despite the edges also being attractive to deer in terms of resources. Similarly, we found low pellet density, indicating lower deer activity in smaller plots (49 and 200 m^2^) that received the same level of treatments as larger plots. This suggests that factors other than food quality and availability, most likely predation risk, are influencing the foraging behaviour of deer. Quantifying predation risk and associated behavioural responses of prey is a challenging task.

We used pellet density as a proxy to quantify the level of use of a given area by deer, assuming that the presence of pellet groups indicates that animals have visited and utilised the area. However, pellet groups do not provide information on the number of animals using the area, the extent of their use or the activities they are performing. GPS‐collar and camera traps are widely used to quantify predation risk and prey response in carnivore‐ungulate systems (Moll et al., 2016; Prugh et al., 2019), although they also do not directly count the number of individuals. Emerging and advanced technologies, such as drones and GPS video collars, have the potential to provide more comprehensive insights by enabling precise animal counts and detailed observations of behaviour and movement patterns (Eikelboom et al., 2019; Kuijper et al., 2013; Prugh et al., 2019; Yu et al., 2023). While our study has limitations, including the use of pellet density as a proxy, our findings underscore the importance of integrating the ‘landscape of fear [or risk]’ concept into habitat management strategies to optimise wildlife conservation efforts.

### 4.1 Management implications

Applying insights from the ‘landscape of fear’ (or, better, ‘landscape of risk’) concept and our experimental results to the management of subtropical monsoon grasslands could yield a novel approach with many opportunities to enhance ecologically well‐reasoned interactions between predators and their prey populations (cf. Gaynor et al., 2021). An important consideration is our advocacy for a ‘soft approach,’ which utilises insights gained from animal behaviour and their use of space, instead of a ‘hard approach’ based on shooting and killing. A soft approach aligns better with many cultural norms and values in South and East Asia (Harvey, 2007; Knight, 2004; Phelps, 2004). This approach also tends to be more sustainable, fostering natural predator–prey dynamics and promoting long‐term minimal interference. Thus, we would favour reducing the number of deer, if desired, by increasing the predation rate by tigers, or reducing the number of tigers, if desired, by making the deer less easy to catch.

Landscape features and habitat structures play a crucial role in determining the level of perceived predation risk, influencing both the behaviour of predators and prey (Gaynor et al., 2019). The hunting success rate of ambush predators like tigers increases in the area with dense vegetation cover (Karanth & Sunquist, 2000; Sunquist, 2010). Conversely, for cursorial predators like wolves and wild dogs, dense vegetation can hinder their hunting success, while open habitats facilitate it (Lone et al., 2014). Similarly, prey species that prefer to hide require dense vegetation cover, whereas those that prefer to flee benefit from open areas (Chamaillé‐Jammes et al., 2019; Gorini et al., 2011). Additionally, prey adjust their habitat use based on the diurnal or nocturnal activity of predators. Prey, especially herbivores, continuously adjust their use of the landscape in response to spatio‐temporal changes in risk (Chamaillé‐Jammes et al., 2019). In line with these dynamics, conservation area managers should devise habitat management strategies that align with the specific landscape features and help maintain delicate prey–predator dynamics.

Conservation area managers can reduce predation risk for deer in high‐risk areas in these tropical monsoon grasslands by creating open areas or refuges for prey species by mowing or by judicial small‐scale, well‐controlled burning in which artificial fertilisation can be carried out to enhance attraction for deer. Our results, and those of others, show that there is a scale effect in operation: for ungulates with different body sizes, patches of vegetation should be kept open and free of obstacles behind which predators can lurk, with a diameter of at least 30–40 m (Elliott et al., 1977). This likely results in the aggregation of deer, which often translates into discernible vegetation patterning (Ford et al., 2014; Schmitz, 2008; Schmitz et al., 2004). This can have wanted or unwanted effects on biodiversity, but it is a tool that managers have at their disposal. Current grassland management practice often involves large‐scale burning and mowing to create open areas covering the entire grassland patches. This approach can result in insufficient grazing pressure from an existing assemblage of herbivores in the area, allowing grasses to grow (Thapa et al., 2022). Consequently, intensive management is required in the following season to ensure the availability of quality forage to the herbivores.

Our results also showed that pellet density is directly related to the spatial extent of openness and grass height, and to a lesser extent to the quality of the forage, but these factors are also modulated by the level of predation risk (Gaynor et al., 2019). Stalking predators like tigers may be at a disadvantage in open and visible areas because their hunting success rate is reduced (Karanth & Sunquist, 2000; Sunquist, 2010). This poses a conundrum for park managers: how to manage the habitat in the park so that both predators and prey may benefit? In low predation‐risk areas for deer, interventions such as increasing vegetation cover or creating water sources can attract predators and hence increase predation risk. Creating small ditches and dikes or dragging logs into open spaces may increase the risk in such landscapes, allowing managers to shift grazing and browsing away from these areas if, for instance, the regeneration of vegetation is desired. Likewise, in a high‐risk area, park manager can clear surrounding tall grasses and bushes to increase visibility thereby reducing predation risk. By integrating ‘landscape of fear’ concept into management, park managers can influence how much herbivores feel at ease, and therefore, how long they stay in a patch foraging and how much they can focus on foraging rather than being vigilant. This likely results in the shifting of grazing and browsing which often translates into discernible vegetation patterning (Ford et al., 2014; Schmitz, 2008; Schmitz et al., 2004). Vegetation patterning is not only a result of differential predation pressure in the landscape (Kuijper et al., 2013; van Ginkel et al., 2019), but it can also be generated by management and factors like environmental (microclimate) and geographical (distance to water and distance to settlements/road) factors.

Consideration should be given to addressing the ecological requirements of both predators and prey while integrating the ‘landscape of fear’ concept into management strategies. Creating small grazing lawns in subtropical monsoon grasslands where grass can grow over 2 m high (Lehmkuhl, 1994; Peet et al., 1999; Thapa et al., 2021) and easily conceal tigers is a waste of time and effort if the aim is to increase the number of deer in an area with large predators like tigers. Grazing lawns will not form because grazers will avoid such areas (Thapa et al., 2021, 2023). Conversely, creating large open areas would harm tigers since it reduces their chances of catching prey by making them too vulnerable. It is unlikely that grazing lawns could form in large open areas if deer numbers are not high enough to exert a high grazing pressure (Thapa et al., 2021, 2022), demanding resources for continuous interventions.

Hence, we believe that creating and maintaining mosaics of 1–2 ha patches (as shown in Figure 1) of short grasses (10–15 cm height) within tall monsoon grasslands would benefit small‐ and medium body‐sized grazers viz., chital and swamp deer by allowing them to optimise the trade‐off between risk and resources. This may reinforce the grazing feedback for culminating in herbivore‐dominated state (Thapa et al., 2022; Venter et al., 2017). Such herbivore‐dominated state would promote a grazing‐tolerant herbaceous layer characterised by low‐stature growth form with higher forage quality, making the area attractive for grazing and thereby facilitating the formation and maintenance of grazing lawns (Thapa et al., 2023). This eventually will ensure maximum survival for the deer that are to be preyed upon by tigers (Thapa et al., 2021). Therefore, it is recommended that these interventions be carried out continuously for 2–3 years to effectively establish and maintain the herbivore‐dominated state. Additionally, this approach of maintaining mosaics not only benefits deer but also provides refuge habitats for other grassland dependents small mammals, herpetofauna and grassland‐dependent birds (Poudyal et al., 2008) including endangered Bengal florican (*Houbaropsis bengalensis*). More importantly, this approach must benefit tigers and help keep them within the national park boundaries, thereby preventing conflicts with people. Determining the optimal size and arrangement of these patches to benefit both predators and prey is a complex task. While ecologists can provide essential scientific insights, it is the skill and expertise of park managers that are crucial in navigating this complexity. Through iterative and adaptive management practices, park managers can play a key role in discovering, implementing and refining strategies that balance the needs of both predators and prey.

## 5 CONCLUSION

The overwhelming success of tiger conservation in Nepal and a subsequent increasing number of incidents of human–tiger conflicts (Fitzmaurice et al., 2021) stresses the urgency to manage the habitat that is within the park for both the predators and the prey if the authorities in charge are to maintain the sizeable tiger population for the future generation. The challenge the Government of Nepal is to entice the estimated 355 adult tigers (DNPWC & DFSC, 2022) and their offspring to stay in the unfenced national parks at the numbers that have been achieved through dedicated protection and to maintain the cervid prey base at its level to feed those tigers. What we thus seek is science‐based management interventions that exclude killing of tigers, but where habitat management (i.e. mowing, burning, fertilising; perhaps logging and uprooting of woody perennials to create open patches) is now becoming permissible for the management authority. The scientific underpinning of the ‘landscape of risk’ concept fundamentally addresses this interaction between predators, prey and vegetation in a spatial context. With the increasing trend of degradation of grassland habitats in the subtropical region of the Indian subcontinent (Ratnam et al., 2016; Sankaran, 2005) and a consequent threat of local extinction of globally threatened faunal species, we posit important conservation implications of our findings.

## AUTHOR CONTRIBUTIONS

**Shyam Kumar Thapa:** Conceptualization (equal); data curation (lead); formal analysis (lead); methodology (lead); project administration (equal); writing – original draft (lead); writing – review and editing (equal). **Joost F. de Jong:** Conceptualization (equal); formal analysis (supporting); methodology (supporting); validation (equal); writing – review and editing (equal). **Anouschka R. Hof:** Conceptualization (equal); methodology (equal); validation (equal); writing – review and editing (equal). **Naresh Subedi:** Conceptualization (equal); funding acquisition (equal); methodology (equal); project administration (equal); validation (equal); writing – review and editing (equal). **Yorick Liefting:** Methodology (equal); software (equal); visualization (equal); writing – review and editing (equal). **Herbert H. T. Prins:** Conceptualization (equal); formal analysis (equal); funding acquisition (lead); project administration (lead); resources (equal); supervision (lead); writing – review and editing (equal).

## CONFLICT OF INTEREST STATEMENT

The authors declare that they have no conflict of interest.

## Supporting information


Figure S1. and S2.

## Data Availability

The data that support the findings of this study are openly available in the Mendeley Data repository: https://data.mendeley.com/preview/558b4hphwm?a=54c9b44c‐0ad1‐47f2‐b240‐4ee06493a427.

## APPENDIX A

### A.1

Results of the linear mixed effects model on the effect of mowing, fertilisation, plot size, species, season and vegetation height on the level of use of the experimental plots by the three deer species.

**Table:**

Treatments	Sum of squares	Mean squares	*df*	*F* value	*p* Value
**Mowing**	**0.20**	**0.10**	**2**	**29.65**	**<.001*****
**Fertilisation**	**0.04**	**0.02**	**2**	**6.26**	**.002****
**Plot size**	**1.13**	**0.57**	**2**	**169.71**	**<.001*****
**Species**	**12.00**	**6.00**	**2**	**1799.23**	**<.001*****
**Season**	**0.60**	**0.30**	**2**	**89.42**	**<.001*****
**Mowing × Fertilisation**	**0.06**	**0.02**	**4**	**4.54**	**.001****
**Mowing × Plot size**	**1.06**	**0.27**	**4**	**79.64**	**<.001*****
**Fertilisation × Plot size**	**0.05**	**0.01**	**4**	**3.53**	**.007****
**Mowing × Species**	**4.19**	**1.05**	**4**	**313.98**	**<.001*****
**Fertilisation × Species**	**0.04**	**0.01**	**4**	**3.09**	**.015***
**Plot size × Species**	**1.38**	**0.35**	**4**	**103.60**	**<.001*****
**Mowing × Season**	**0.15**	**0.04**	**4**	**10.96**	**<.001*****
Fertilisation × Season	0.01	0.00	4	0.47	.76
Plot size × Season	0.02	0.00	4	1.23	.29
**Species × Season**	**1.68**	**0.42**	**4**	**125.88**	**<.001**
Mowing × Fertilisation × Plot size	0.04	0.01	8	1.54	.14
Mowing × Fertilisation × Species	0.04	0.01	8	1.59	.12
**Mowing × Plot size × Species**	**1.34**	**0.17**	**8**	**50.12**	**<.001**
**Fertilisation × Plot size × Species**	**0.06**	**0.01**	**8**	**2.34**	**.02***
Mowing × Fertilisation × Season	0.04	0.00	8	1.38	.2
Mowing × Plot size × Season	0.04	0.00	8	1.32	.23
Fertilisation × Plot size × Season	0.01	0.00	8	0.41	.92
**Mowing × Species × Season**	**0.26**	**0.03**	**8**	**9.83**	**<.001*****
Fertilisation × Species × Season	0.02	0.00	8	0.76	.63
Plot size × Species × Season	0.01	0.00	8	0.47	.88
Mowing × Fertilisation × Plot size × Species	0.07	0.00	16	1.36	.15
Mowing × Fertilisation × Plot size × Season	0.03	0.00	16	0.51	.94
Mowing × Fertilisation × Species × Season	0.07	0.00	16	1.40	.13
Mowing × Plot size × Species × Season	0.07	0.00	16	1.23	.24
Fertilisation × Plot size × Species × Season	0.02	0.00	16	0.43	.97
Mowing × Fertilisation × Plot size × Species × Season	0.04	0.00	32	0.41	.99
**Grass average height (co‐variate)**	**0.06**	**0.06**	**1**	**18.06**	**<.001*****

*Note*: Significance codes: **p* < .05, ***p* < .01, ****p* < .001; *p* > .05 = ns.

## APPENDIX B

### B.1

Vegetation height (cm) with respect to treatment. Vegetation height was significantly lower in four times mown plots (*F* = 154.95, *p* < .001) which is evident but, alteration in vegetation height may change risk perception in herbivores through modification in visibility and detection probability. Letters above each boxplot indicate a significant difference at alpha = .05, tested by estimated marginal means after the linear mixed effect model. Groups that share the same letter are not significantly different from each other. We used mowing, and plot size as fixed factors and replications within locations as random factors in the model.

## APPENDIX C

### C.1

Relationship between grass height and level of use (in terms of pellet group density) by deer species. Deer species respond to grass height differently (*F* = 610.09, *p* < .001) indicating, differential risk perception between deer species. Level of use decreases with grass height. Grass height expressed as log^e^ grass height. Regression lines were obtained from liner mixed effect model with height and species as predictor variables and replications within locations as random effect.

## APPENDIX D

### D.1

Results of the linear mixed effect model on the effects of mowing, fertilisation, species and season on the level of use by the three deer species between the edge and the centre of the 3600 m^2^ plots (point in the model).

**Table:**

Treatments	Sum of squares	Mean squares	*df*	*F* value	*p* Value
**Mowing**	0.904	0.904	1	112.3	**<.001*****
**Fertilisation**	0.249	0.125	2	15.48	**<.001*****
**Species**	24.571	12.285	2	1526.26	**<.001*****
**Season**	0.742	0.371	2	46.12	**<.001*****
**Point**	1.381	1.381	1	171.55	**<.001*****
Mowing × Fertilisation	0.002	0.001	2	0.15	.857
**Mowing × Species**	2.095	1.048	2	130.14	**<.001*****
**Fertilisation × Species**	0.262	0.066	4	8.14	**<.001*****
Mowing × Species	0.047	0.023	2	2.91	.055
Fertilisation × Season	0.026	0.007	4	0.82	.513
**Species × Season**	1.446	0.362	4	44.92	**<.001*****
**Mowing × Point**	0.142	0.142	1	17.67	**<.001*****
Fertilisation × Point	0.034	0.017	2	2.13	.12
**Species × Point**	1.59	0.795	2	98.79	**<.001*****
**Season × Point**	0.195	0.097	2	12.11	**<.001*****
Mowing × Fertilisation × Species	0.018	0.004	4	0.56	.695
Mowing × Fertilisation × Season	0.018	0.005	4	0.56	.689
Mowing × Species × Season	0.044	0.011	4	1.37	.242
Fertilisation × Species × Season	0.068	0.009	8	1.06	.391
Mowing × Fertilisation × Point	0.005	0.002	2	0.29	.748
**Mowing × Species × Point**	0.157	0.078	2	9.73	**<.001*****
Fertilisation × Species × Point	0.03	0.008	4	0.94	.438
**Mowing × Season × Point**	0.059	0.029	2	3.65	**.027***
Fertilisation × Season × Point	0.001	0	4	0.04	.997
**Species × Season × Point**	0.101	0.025	4	3.13	**.0146***
Mowing × Fertilisation × Species × Season	0.062	0.008	8	0.96	.4672
Mowing × Fertilisation × Species × Point	0.003	0.001	4	0.08	.9877
Mowing × Fertilisation × Season × Point	0.004	0.001	4	0.14	.9678
Mowing × Species × Season × Point	0.061	0.015	4	1.9	.1095
Fertilisation × Species × Season × Point	0.012	0.002	8	0.19	.9922
Mowing × Fertilisation × Species × Season × Point	0.023	0.003	8	0.35	.9444

*Note*: Significance codes: **p* < .05, ****p* < .001.
