# Supplementary material for: Integration of the landscape of fear concept in grassland management: An experimental study on subtropical monsoon grasslands in Bardia National Park, Nepal
Source: Ecol Evol. 2024 Aug 1;14(8):e70098. doi: 10.1002/ece3.70098 (PMC11294578; doi:10.1002/ece3.70098)
Supplement: Supplementary file 1 — Figure S1. and S2. [file ECE3-14-e70098-s001.docx]

**Supplementary Materials for:**

**Integration of the landscape of fear concept in grassland management – an experimental study on subtropical monsoon grasslands in Bardia National Park, Nepal.**

Shyam Kumar Thapa ^a, 1, *^, Joost F. de Jong ^b, 2^, Anouschka R. Hof ^b, 3^, Naresh Subedi ^a, 4^, Yorick Liefting ^b, 5^, and Herbert H.T. Prins ^c, 6^

^a^ National Trust for Nature Conservation, PO Box 3712, Khumaltar, Lalitpur 44700, Lalitpur, Nepal.

^b^ Wildlife Ecology and Conservation Group, Wageningen University and Research, Droevendaalsesteeg 4, 6708 PB Wageningen, The Netherlands.

^c^ Animal Sciences Group, Wageningen University, De Elst 1, 6708 WD, Wageningen, The Netherlands.


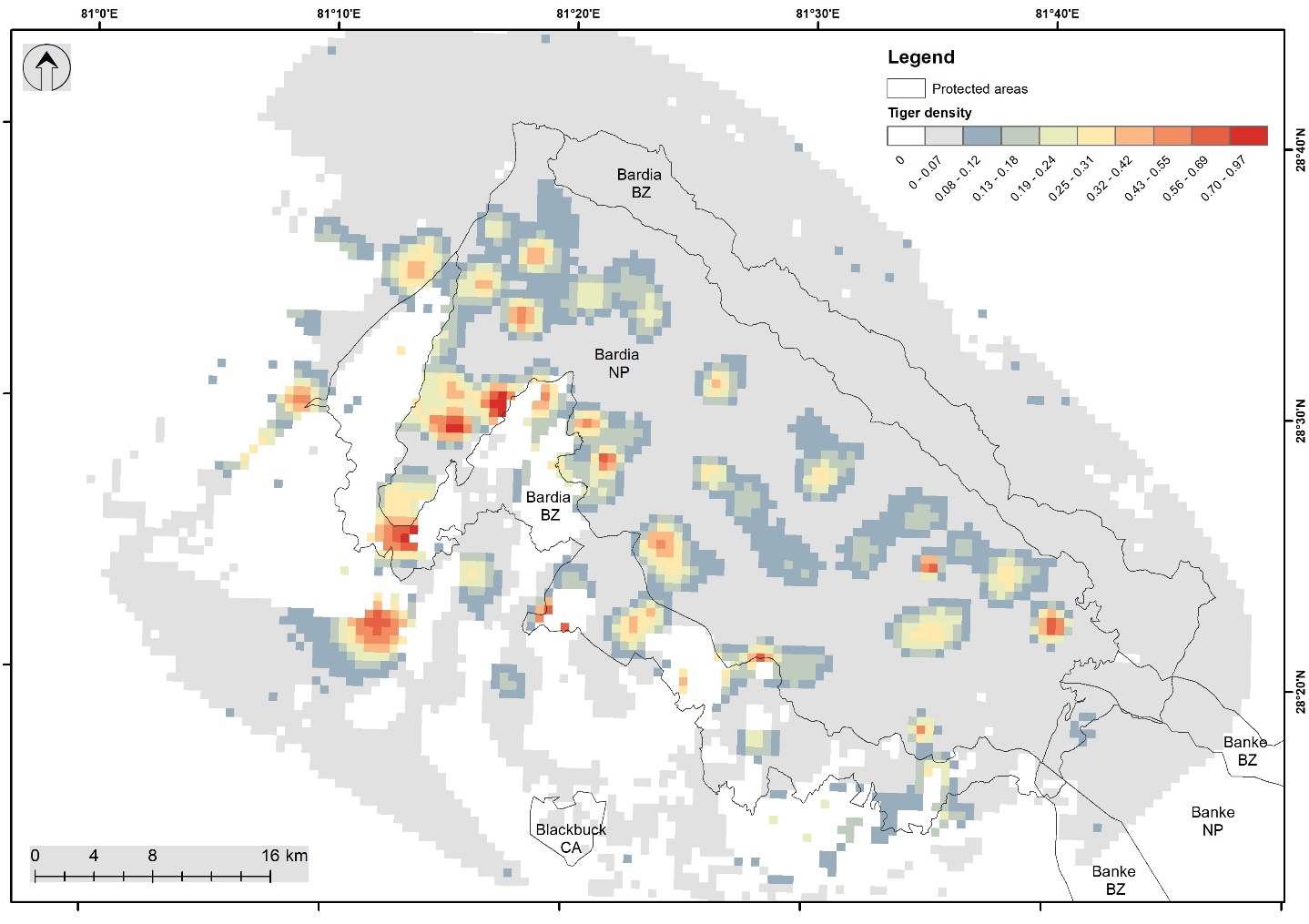


Experimental sites

**Figure S1**: Tiger density map of Bardia National Park (DNPWC and DFSC 2022). Density of tiger in Bardia National Park has increased from 4.74 individuals. 100 km^-2^ in 2018 to ~7.15 individuals. 100 km^-2^ in 2022 (DNPWC and DFSC 2022). The experimental sites fall within the area with the highest density of tigers in the Bardia NP.

**Edge**

**Edge**

**Centre**

**15 m**

**15 m**

**15 m**

**15 m**

**15 m**

**60 m**

**60 m**

**15 m**

**15 m**

**15 m**

**15 m**

**Figure S2:** Spatial layout of sampling points in 3600 m^2^ plots. Each sampling point (indicated by small squares – measuring 2 m x 2m) is 15 m in distance from each other and also from the corner of the plot. The edge area of the plot is denoted by a grey shed and the central area is by white with grey dots.
